# Uncovering hidden diversity in *Didymellaceae* from Yunnan: Introducing three novel species of *Didymella* and *Nothophoma*

**DOI:** 10.3897/mycokeys.141.205517

**Published:** 2026-10-08

**Authors:** Rungtiwa Phookamsak, Hongbo Jiang, Junfu Li, Putarak Chomnunti, Haolun Qi, Darbhe Jayarama Bhat, Jaturong Kumla, Jianchu Xu, Laith Khalil Tawfeeq Al-Ani, Saisamorn Lumyong, Nakarin Suwannarach

**Affiliations:** 1 Department of Economic Plants and Biotechnology, Yunnan Key Laboratory for Wild Plant Resources, Kunming Institute of Botany, Chinese Academy of Sciences, Kunming 650201, Yunnan Province, China Department of Economic Plants and Biotechnology, Yunnan Key Laboratory for Wild Plant Resources, Kunming Institute of Botany, Chinese Academy of Sciences Kunming China; 2 Center of Excellence in Microbial Diversity and Sustainable Utilization, Faculty of Science, Chiang Mai University, Chiang Mai, Thailand Center of Excellence in Microbial Diversity and Sustainable Utilization Chiang Mai Thailand; 3 Honghe Center for Mountain Futures, Kunming Institute of Botany, Chinese Academy of Sciences, Honghe, China Honghe Center for Mountain Futures, Kunming Institute of Botany, Chinese Academy of Sciences Honghe China; 4 Department of Botany and Microbiology, College of Science, King Saud University, P.O. Box 2455, Riyadh 11451, Saudi Arabia Department of Botany and Microbiology, College of Science, King Saud University Riyadh Saudi Arabia; 5 Vishnugupta Vishwavidyapeetam, Ashoke, Gokarna 581326, India Vishnugupta Vishwavidyapeetam Gokarna India; 6 Office of Research Administration, Chiang Mai University, Chiang Mai, 50200, Thailand Office of Research Administration, Chiang Mai University Chiang Mai Thailand; 7 Department of Biology, Faculty of Science, Chiang Mai University, Chiang Mai 50200, Thailand Department of Biology, Faculty of Science, Chiang Mai University Chiang Mai Thailand; 8 Centre for Mountain Futures (CMF), Kunming Institute of Botany, Kunming, China Centre for Mountain Futures (CMF), Kunming Institute of Botany Kunming China; 9 CIFOR-ICRAF China Program, World Agroforestry (ICRAF), Kunming, China CIFOR-ICRAF China Program, World Agroforestry (ICRAF) Kunming China; 10 School of Biology Science, Universiti Sains Malaysia, 11800 Minden, Pulau Pinang, Malaysia School of Biology Science, Universiti Sains Malaysia Minden Malaysia; 11 Academy of Science, The Royal Society of Thailand, Bangkok 10300, Thailand Academy of Science, The Royal Society of Thailand Bangkok Thailand

**Keywords:** *
Dothideomycetes
*, morpho-molecular approach, multi-locus phylogeny, *
Pleosporales
*, saprobic fungi, species novelty, taxonomy

## Abstract

Ascomycete diversity in Yunnan Province, China, remains substantially underexplored. Many species likely remain undetected. This study aimed to discover the ascomycete diversity in Yunnan, China, depending on three fungal collections obtained from Baoshan and Kunming city regions. Based on morphological characteristics, host association, ecological habitats, and multi-locus phylogenetic analyses of these collections, together with representative strains of taxa in *Didymella* and *Nothophoma*, three new species are introduced, namely *Didymella baoshanensis*, *Nothophoma aceris*, and *N. ginkgonis*. Phylogenetic analyses of a concatenated ITS, LSU, *rpb2*, and *tub2* sequence dataset using maximum likelihood and Bayesian inference methods supported the novelty of these taxa within *Didymella* and *Nothophoma*. Phylogenetically, *D. baoshanensis* forms a distinct sister lineage to the type strain of *D. brevipilosa*, with strong statistical support. *Nothophoma aceris* clusters with the invalidly published name *N. juglandis* with high support values. While *N. ginkgonis* forms a distinct branch as basal of *N. ferruginea*, *N. platycladi*, and *N. pruni*. *Didymella baoshanensis* was found as a saprobe on dead fern rachises and is characterized by multi-loculate ascostromata; subglobose to ampulliform, ostiolate ascomata; 8-spored, bitunicate, fissitunicate, cylindrical to subcylindrical-clavate asci embedded in broadly cellular pseudoparaphyses; and fusiform, hyaline, 1-septate ascospores. *Nothophoma aceris* was found as a saprobe on dead aerial twigs of *Acer palmatum* and is characterized by scattered to clustered, globose to subglobose, stromatic, brown to dark brown, glabrous, ostiolate, apapillate, pycnidial conidiomata; holoblastic, phialidic conidiogenous cells, sometimes proliferating at the same level, with 1–2 conidiogenous loci; and hyaline to brown or dark brown, subglobose, ovoid to obovoid, aseptate, thick- and smooth-walled conidia. *Nothophoma ginkgonis* was found as a saprobe on dead aerial twigs of *Ginkgo biloba* and is characterized by subglobose to hemispherical or ellipsoidal, uni-loculate, brown to dark brown, glabrous, ostiolate, apapillate, pycnidial conidiomata; holoblastic, phialidic, hyaline, ampulliform to somewhat isodiametric, unbranched, determinate, discrete, smooth-walled conidiogenous cells; and hyaline to light brown, or yellowish-brown, subglobose to ellipsoidal, or obovoid, aseptate, smooth-walled conidia. Detailed description, photographic illustration, and updated phylogenetic analyses of *Didymella* and *Nothophoma* are provided in the present study.

## Introduction

*Didymella* was first designated as the generic type of *Didymellaceae* by Saccardo (1880) with *D. exigua* as the type species. Later Chen et al. (2015) emended *Peyronellaea* with *Didymella* and synonymized several species, previously identified as *Phoma* to the genus based on multi-locus phylogenetic analyses of a combined ITS, LSU, *rpb2*, and *tub2* sequence dataset. There are more than 500 species epithets listed under *Didymella* in Index Fungorum (2026) and MycoBank (Robert et al. 2005). Despite this large number of recorded names, the taxonomy and diversity of *Didymella* remain relatively poorly known. Less than half of the known species have so far been clarified based on molecular phylogeny and sexual-asexual morphological characteristics (de Gruyter et al. 2009, 2013; Aveskamp et al. 2010; Chen et al. 2015, 2017, 2023; Valenzuela-Lopez et al. 2018; Hou et al. 2020a; Wijayawardene et al. 2020).

*Didymella* has a long and complex taxonomic history, and species delineation remains problematic (de Gruyter et al. 2009; Chen et al. 2015, 2017; Hou et al. 2020a). Saccardo (1878) first recognized the genus as “Didymosphaeria (Didymella) culmigena” and treated *Didymella* as a subgenus of *Didymosphaeria* on the basis of *Didymosphaeria exigua*. Consequently, Saccardo (1880) erected *Didymella* to the generic level with the description of *Didymella exigua* (≡ *Didymosphaeria exigua*). However, de Gruyter et al. (2009) suggested that the genus should be validated to Saccardo (1882), when a Latin diagnosis was provided. The taxonomic affinity of *Didymella* has been discussed and replaced by various authors in 19^th^ to early 20^th^ century, mainly based on morphological characteristics. Saccardo (1880) initially listed the genus in *Sphaeriaceae* (Illegitimate Art. 18.1; Index Fungorum 2026) under “section 4 Didymosporae” on the basis of *D. exigua*. De Gruyter et al. (2009), on the other hand, noted that the genus was originally placed in *Mycosphaerellaceae (Mycosphaerellales)* together with *Mycosphaerella*. Subsequently, the genus was transferred to many different families, including *Phaeosphaeriaceae* (Barr 1979; Silva-Hanlin and Hanlin 1999), *Pleosporaceae* (Sivanesan 1984), and *Venturiaceae* (Reddy et al. 1998), as well as *Pleosporales* genus *incertae sedis* (Lumbsch and Huhndorf 2007). De Gruyter et al. (2009) designated a neotype of *D. exigua* [dried culture CBS H-20123, culture ex-neotype CBS 183.55, *ex Rumex arifolius* (*Polygonaceae*) France] because the holotype material of *Didymosphaeria exigua* could not be located in either M or BRNU, where the main original collections of Niessl von Mayendorf were preserved. De Gruyter et al. (2009) re-circumscribed the genus based on morphological study of the neotype and molecular data and hence, established the family *Didymellaceae* to accommodate *Didymella* and another sexual genus *Leptosphaerulina*, corresponding with other asexual genera. Besides, the phylogenetic analyses inferred by de Gruyter et al. (2009) also demonstrated that *Ascochytasensu stricto* was related to *Didymella*. Consequently, Aveskamp et al. (2010) also linked *Phomasensu stricto* to *Didymella*. While Chen et al. (2015) treated *Ascochyta*, *Didymella* and *Phoma* as distinct genera based on the evidence of multi-locus phylogenetic analyses that each genus restricted to a monophyletic group. These three genera were re-circumscribed and generic emended together with the genus *Epicoccum* whereas *Microsphaeropsis* was raised to the familial rank as *Microsphaeropsidaceae* due to the morphological distinctiveness and phylogenetic placement as basal to the *Didymellaceae* (Chen et al. 2015). Nevertheless, *Microsphaeropsidaceae* was currently treated as a synonym of *Didymellaceae* (Hyde et al. 2024). Since the family was revisited by Chen et al. (2017), the number of recognized *Didymella* species has increased rapidly. More than 35 novel species have been introduced to the genus based on a polyphasic approach (Index Fungorum 2026). However, more than 200 traditional species of *Didymella* established during the 18^th^ to 19^th^ centuries remain taxonomically unresolved. Hence, an ascribed morphological-molecular approach based on type material is still needed to resolve the phylogenetic affinities of these poorly known species.

*Nothophoma*, belonging to family *Didymellaceae*, was established by Chen et al. (2015) to accommodate phoma-like taxa and is typified by *N. infossa* (≡ *Phoma infossa*; ex-neotype CBS 123395). Chen et al. (2015) initially included five species in the genus, namely *N. anigozanthi*, *N. arachidis*-*hypogaeae*, *N. quercina*, *N. gossypiicola*, and *N. infossa*. These five species were historically classified within *Phoma* but formed a distinct monophyletic clade separate from *Phomasensu stricto*. Chen et al. (2015) also noted that the genus exhibits a degree of host-specificity. For instance, *N. infossa* is consistently associated with ash trees (*Fraxinus* spp.), whereas *N. gossypiicola* has only been found on cotton plants (*Gossypium* spp.) (Aveskamp et al. 2010; Chen et al. 2015). Subsequently, 21 additional species were accommodated within the genus, which occurred on various plant hosts as saprobes or pathogens, but some species have also been found from human bronchial wash samples (Crous et al. 2016, 2017; Abdel-Wahab et al. 2017; Valenzuela-Lopez et al. 2018; Chethana et al. 2019; Hou et al. 2020a, 2020b; Mapook et al. 2020; Zhang et al. 2020, 2021; Keirnan et al. 2021; Jiao et al. 2024; Zhao et al. 2024; Guo et al. 2025). There are 26 epithets listed in Index Fungorum (2026). Although *Nothophoma* was introduced relatively recently, most of its species are well-justified based on a morpho-molecular approach. However, some *Nothophoma* species taxonomy remains uncertain due to unresolved phylogenetic relationships within the *N. quercina* species complex (Nekrasov et al. 2022).

During the investigation of ascomycete diversity in Yunnan Province, China, saprobic fungal specimens were collected. Consequently, the objective of this study is to describe and introduce the novel species within the genera *Didymella*, and *Nothophoma* based on an integrated morphological and molecular approach.

## Materials and methods

### Sample collection, isolation and morphological examination

The samples were collected from Baoshan and Kunming (Yunnan, China) regions, during 2015 to 2017, and brought to the mycological laboratory in paper bags for further observation and isolation. The new isolates were discovered on dead rachis of a fern, and dead aerial twigs of *Acer palmatum* (*Sapindaceae*) and *Ginkgo biloba* (*Ginkgoaceae*). Ascomata/conidiomata visualized on the host surface were observed under an Olympus SZ61 stereomicroscope (Model SZ2-ILST; Olympus Corporation, Tokyo, Japan) and photographed by a digital camera. Micro-morphological features were examined under a Nikon ECLIPSE Ni-U compound microscope with DIC microscopy and images captured with a Nikon DS-Ri2 camera (Nikon Instruments Inc., Tokyo, Japan). Mucilaginous sheaths or appendages surrounding ascospores/ conidia were observed by staining with India ink. Congo red and Melzer’s reagent were used to observe the ascomal/pycnidial centrum (e.g., pseudoparaphyses, ascus apical apparatus, and ascospore characters). Measurements of morphological features were made with Tarosoft (R) Image Frame Work program. The photomicrographic pictures of morphological characteristics were edited and combined using Adobe Photoshop CS6 Extended version 10.0 software (Adobe Systems Inc., San Jose, CA, USA). The specimen was dried with silica gel in a paper bag for preservation. The fungarium specimens were deposited at the Herbarium of Cryptogams Kunming Institute of Botany Academia Sinica (KUN-HKAS), China.

Pure fungal cultures were obtained via single spore isolation using the spore suspension technique (Senanayake et al. 2020). Five germinated spores, with two replicates, were transferred to sterilized potato dextrose agar (PDA; 39 g/l sterile distilled water, OXOID^TM^ potato dextrose agar) plate, incubated at 25–27 °C for 1–2 weeks and subsequently subcultured onto PDA. The fungal colony characteristics and growth were observed within 10 days while the sporulation *in vitro* was observed at 4–8 weeks after cultivation at 25–27 °C in the dark. The pure culture was preserved in PDA for short-term storage and in sterilized distilled water and 10% glycerol for long-term storage. Pure fungal cultures were deposited at the Culture Collection of Kunming Institute of Botany (KUNCC/ KUMCC; formerly used codes), Kunming, China. Index Fungorum numbers were registered for the new species (http://indexfungorum.org/Names/IndexFungorumRegisterName.asp; accessed on 26 June 2026). The justification of the novelty of fungi was as followed by Jayawardena et al. (2021) and Maharachchikumbura et al. (2021).

### DNA extraction, PCR amplification and sequencing

Fresh mycelia were cultivated on PDA for 4 weeks, after which approximately 50 mg of mycelium was scraped and transferred to a sterilized 1.5 ml microcentrifuge tube for DNA extraction using the Biospin Fungus Genomic DNA Extraction Kit (BioFlux®, Hangzhou, P.R. China). DNA amplification was performed by polymerase chain reaction (PCR) using primers specific to the target loci. The primer pairs ITS5/ITS4 (White et al. 1990), LR0R/LR5 (Vilgalys and Hester 1990; Rehner and Samuels 1994), Btub2Fd and Btub4Rd (Woudenberg et al. 2009) and fRPB2-5F and fRPB2-7cR (Liu et al. 1999), were used to amplify the internal transcribed spacers region of ribosomal DNA (ITS; ITS1-5.8S-ITS2), the 28S large subunit nuclear ribosomal DNA (LSU), the partial beta-tubulin gene (*tub2*) and the RNA polymerase II second largest subunit (*rpb2*), respectively. Owing to differences in the primer sets used for amplification of the *tub*2 and *rpb*2 loci among previously published *Nothophoma* taxa and, in some cases, the quality of the assembled sequences, some taxa may contain only short overlapping regions in the sequence alignments. Consequently, direct nucleotide comparisons for these loci were not possible for certain taxa.

The total volume of PCR components was 25 μL, containing 2 μL of DNA template (30–50 ng/μL), 1 μL of each forward and reverse primer, 12.5 μL of PCR MasterMix (mixture of *EasyTaq*TM DNA Polymerase, dNTPs, and optimized buffer; Beijing TransGen Biotech Co., Ltd., Chaoyang District, Beijing, China) and 8.5 μL of the sterilized double-distilled water (ddH_2_O). The PCR thermal cycling of these genes was set according to the protocols described by Chen et al. (2015) and Li et al. (2024). PCR products were sent to TsingKe Biological Technology, Kunming City, Yunnan Province, China for purification and sequencing.

### Phylogenetic analyses

Consensus sequences were assembled by BioEdit 7.1.3.0 (Hall 1999) and compared using nucleotide BLAST searches against the GenBank database (www.ncbi.nlm.nih.gov/blast/) for preliminary identification and selection of closely related taxa as well as locating the target group studies. Reference sequences were retrieved from the nucleotide BLAST search results and relevant publications (Hou et al. 2020a; Chen et al. 2023; Wang et al. 2024; Tables 1, 2). Individual gene datasets of ITS, LSU, *rpb2* and *tub2* regions were aligned using MAFFT v.7 (http://mafft.cbrc.jp/alignment/server/index.html; Katoh et al. 2019). Ambiguous sites at the beginning and end of the alignment were trimmed and manually edited where necessary with BioEdit 7.1.3.0 (Hall 1999). Aligned individual gene datasets were analyzed by maximum likelihood (ML), for checking the congruence of tree topologies. If the tree topologies were compatible, a concatenated multi-locus sequence dataset (ITS, LSU, *rpb2*, and *tub2*) was further analyzed using maximum likelihood (ML), and Bayesian inference (BI) analyses to determine phylogenetic relationships of the novel species with other related taxa of the genera *Didymella* and *Nothophoma* in *Didymellaceae*.

**Table 1.** Taxa table of *Didymella* species and their corresponding GenBank accession numbers used in the present phylogenetic analyses. Ex-type strains are indicated in black bold and new species is indicated in cells with light grey shading. Unavailable sequences are indicated by n/a.

**Species name**	**Strain number**	**GenBank accession numbers**			
		**ITS**	**LSU**	***rpb*2**	***tub*2**
***D. acetosellae***	**CBS 631.76**	MN973542	MN943749	MT018176	MT005645
***D. acutilobae***	**ANAC-1901**	OQ749980	OQ749982	OQ744072	OQ744070
***D. aeria***	**CGMCC 3.18353**	KY742051	KY742205	KY742137	KY742293
*D. aliena*	CBS 123831	MN972823	n/a	MN983435	MN983841
***D. aloeicola***	**CBS 562.88**	MN973535	MN943742	MT018164	MT005638
*D. americana*	CBS 112525	FJ426975	MN943738	MT018154	FJ427091
***D. anserina***	**CBS 167.42**	MN973530	MN943736	MT018152	MT005633
***D. aquatica***	**CGMCC 3.18349**	KY742055	KY742209	KY742140	KY742297
***D. arachidicola***	**CBS 333.75**	GU237833	GU237996	KT389598	GU237554
***D. aurea***	**CBS 269.93**	GU237818	GU237999	KT389599	GU237557
***D. azollae***	**A1**	MT514913	MT514910	n/a	MT512516
***D. baileyae***	**VIZR 1.53**	MZ265316	MZ265319	MZ272350	MZ272346
***D. baoshanensis***	**KUNCC 23-13566**	PZ575565	PZ575564	n/a	PZ583459
*D. bellidis*	CBS 714.85	GU237904	GU238046	KP330417	GU237586
***D. bischofiae***	**HJAUP C1776**	OR625712	OR625713	OR620208	OR620206
***D. boeremae***	**CBS 109942**	FJ426982	GU238048	KT389600	FJ427097
***D. brevipilosa***	**FMR 17415**	NR185469	OU612372	OU612359	OU612358
***D. brunneospora***	**CBS 115.58**	KT389505	KT389723	KT389625	KT389802
***D. calidophila***	**CBS 448.83**	FJ427059	GU238052	MT018170	FJ427168
***D. camporesii***	**JZB380040**	MN648211	MN640406	MN984253	MN537432
*D. cari*	CBS 144498	MH327826	MH327862	n/a	MH327900
*D. chenopodii*	CBS 128.93	GU237775	GU238055	KT389602	GU237591
***D. chlamydospora***	**YW23-14**	MK836111	MK836109	LC480708	LC482279
***D. chloroguttata***	**CGMCC 3.18351**	KY742057	KY742211	KY742142	KY742299
***D. chromolaenae***	**MFLUCC 17-1459**	MT214363	MT214457	n/a	n/a
***D. clerodendri***	**HJAUP C1698**	OR625709	OR625714	OR620207	OR611942
***D. coffeae-arabicae***	**CBS 123830**	FJ426993	GU238005	KT389603	FJ427104
***D. combreti***	**CBS 137982**	MN973525	MN943731	MT018139	MT005626
***D. corylicola***	**CBS 146357**	MN954301	MN954290	MN958323	MN958333
*D. curtisii*	CBS 251.92	FJ427038	GU238013	MT018131	FJ427148
***D. cylindrica***	**IRAN 3051C**	OK257014	OK257022	OK247736	OK247741
***D. dactylidis***	**CBS 124513**	GU237766	GU238061	MT018173	GU237599
***D. degraaffiae***	**CBS 144956**	MN823444	MN823295	MN824470	MN824618
***D. digitariae***	**KDI036**	PV830646	PV830650	PV835324	PV835328
***D. dimorpha***	**CBS 346.82**	GU237835	GU238068	MT018158	GU237606
***D. ellipsoidea***	**CGMCC 3.18350**	KY742060	KY742214	KY742145	KY742302
*D. eucalyptica*	CBS 377.91	GU237846	GU238007	KT389605	GU237562
***D. exigua***	**CBS 183.55**	GU237794	EU754155	EU874850	GU237525
***D. finnmarkica***	**CBS 145572**	MK876388	MK876429	MK876484	n/a
***D. gardeniae***	**CBS 626.68**	FJ427003	GQ387595	KT389606	FJ427114
***D. gei***	**CGMCC 3.20068**	MT229698	MT229675	MT239095	MT249266
***D. gigantis***	**ANGI-1914**	OQ746335	OQ746315	OQ731406	OQ731404
***D. glomerata***	**CBS 287.76**	FJ427006	MH872750	MN983461	FJ427117
***D. guttulata***	**CBS 127976**	MN973524	MN943730	MT018138	MT005625
***D. heteroderae***	**CBS 109.92**	FJ426983	GU238002	KT389601	FJ427098
***D. ilicicola***	**CGMCC 3.18355**	KY742065	KY742219	KY742150	KY742307
***D. ilseae***	**BRIP 54066a**	PV425967	n/a	PV459752	PV528542
***D. indica***	**CBS 653.77**	MN973534	MN943741	MT018159	MT005637
***D. infuscatispora***	**CGMCC 3.18356**	KY742067	KY742221	KY742152	KY742309
***D. keratinophila***	**CBS 121686**	MN973519	MN943725	MT018133	MT005620
***D. kooimaniorum***	**CBS 144951**	MN823448	MN823299	MN824474	MN824622
*D. lethalis*	LC 8130	KY742069	KY742223	KY742153	KY742311
***D. ligulariae***	**CGMCC 3.20070**	MT229699	MT229676	MT239096	MT249267
***D. longicolla***	**CBS 124514**	GU237767	GU238095	MT018161	GU237622
***D. macrophylla***	**CGMCC 3.18357**	KY742070	KY742224	KY742154	KY742312
*D. macrostoma*	CBS 223.69	GU237801	GU238096	KT389608	GU237623
***D. maydis***	**CBS 588.69**	FJ427086	EU754192	GU371782	FJ427190
***D. microchlamydospora***	**CBS 105.95**	FJ427028	GU238104	KP330424	FJ427138
***D. miitis***	**CBS 443.72**	MN973523	MN943729	MT018137	MT005624
*D. molleriana*	CBS 229.79	GU237802	GU238067	KP330418	GU237605
*D. musae*	CBS 463.69	FJ427026	GU238011	MT018148	FJ427136
***D. naikii***	**PLS3**	OM952211	OM830704	n/a	OM858681
*D. negriana*	CBS 358.71	GU237838	GU238116	KT389610	GU237635
***D. nigricans***	**CBS 444.81**	GU237867	GU238000	MT018146	GU237558
***D. ocimicola***	**CGMCC 3.18358**	KY742078	KY742232	MT018181	KY742320
***D. pedeiae***	**CBS 124517**	GU237770	GU238127	KT389612	GU237642
***D. pinodella***	**CBS 300.53**	MN973526	MN943732	MT018142	MT005627
***D. pinodes***	**CBS 525.77**	GU237883	GU238023	KT389614	GU237572
***D. pittospori***	**HJAUP C1740**	OR625710	OR625711	n/a	OR620205
***D. poaceicola***	**MFLUCC 13-0212**	KX965726	KX954395	KX898364	n/a
*D. pomorum*	CBS 17691	FJ427056	GU238028	KT389618	FJ427166
***D. prolatricolla***	**CBS 126182**	MN973533	MN943740	MT018157	MT005636
***D. prosopidis***	**CBS 136414**	KF777180	KF777232	MT018149	MT005631
***D. protuberans***	**CBS 381.96**	GU237853	GU238029	KT389620	GU237574
***D. pseudomacrophylla***	**MF-12.28**	MH651551	MH651592	PP750299	MH725218
***D. pteridis***	**CBS 379.96**	KT389504	KT389722	KT389624	KT389801
***D. qilianensis***	**CGMCC 3.20071**	MT229701	MT229678	MT239098	MT249269
*D. rhei*	CBS 109177	GU237743	GU238139	KP330428	GU237653
***D. rosea***	**BRIP 50788**	KT338640	KT287003	n/a	KT286945
***D. rumicicola***	**CBS 683.79**	KT389503	KT389721	KT389622	KT389800
***D. sancta***	**CBS 281.83**	FJ427063	GU238030	MT018132	MT005619
***D. segeticola***	**CGMCC 3.17489**	KP330443	KP330455	KP330414	KP330399
*D. senecionicola*	CBS 160.78	GU237787	GU238143	MT018177	GU237657
***D. sinensis***	**CGMCC 3.18348**	KY742085	KY742239	MT018127	KY742327
***D. subglobispora***	**CBS 364.91**	MN973531	MN943737	MT018153	MT005634
*D. subglomerata*	CBS 110.92	FJ427080	GU238032	KT389626	FJ427186
***D. subherbarum***	**CBS 250.92**	GU237809	GU238145	MT018162	GU237659
***D. subrosea***	**CBS 733.79**	MN973540	MN943747	MT018174	MT005643
***D. suiyangensis***	**CGMCC 3.18352**	KY742089	KY742243	KY742168	KY742331
*D. tabebuiaicola*	LM1190	MZ703619	MZ703624	MZ712361	MZ712365
***D. tanaceti***	**BRIP 50785**	KT338641	KT287022	n/a	KT286974
***D. uniseptata***	**CGMCC 3.20069**	MT229702	MT229679	MT239099	MT249270
***D. variabilis***	**CBS 254.79**	MN973544	MN943751	MT018182	MT005647
*D. viburnicola*	CBS 523.73	GU237879	GU238155	KP330430	GU237667
***D. yenwuae***	**BRIP 15859a**	PQ279215	n/a	PQ299736	PQ299737
***D. yunnanensis***	**CGMCC 3.24241**	OP647939	OP836939	OP854286	OP854551
***Epicoccum brahmanense***	**CBS 990.95**	MN973514	MN943720	MT018120	MT005614

**Table 2.** Taxa table of *Nothophoma* species and their corresponding GenBank accession numbers used in the present phylogenetic analyses. Ex-type strains are indicated in black bold and new species is indicated in cells with light grey shading. Unavailable sequences are indicated by n/a.

**Species name**	**Strain number**	**GenBank accession numbers**			
		**ITS**	**LSU**	***rpb2***	***tub2***
***Nothophoma acaciae***	**CBS 143404**	MG386056	MG386109	MG386144	MG386167
***N. aceris***	**KUNCC 23-13565**	PZ575566	PZ575567	PZ583461	PZ583460
***N. anigozanthi***	**CBS 381.91**	GU237852	GU238039	KT389655	GU237580
***N. arachidis-hypogaeae***	**CBS 125.93**	MH862388	MH874048	KT389656	GU237583
***N. brennandiae***	**CBS 145912**	MN823579	MN823430	MN824604	MN824753
*N. brennandiae*	CBS 125540	MN973555	MN943762	MT018199	MT005658
*N. brennandiae*	CBS 140541	MN973558	MN943765	MT018202	MT005661
*N. brennandiae*	CBS 125539	MN973553	MN943760	MT018197	MT005656
***N. chromolaenae***	**MFLUCC 17-1443**	MT214364	MT214458	n/a	n/a
***N. eucalyptigena***	**CBS 142535**	KY979771	KY979826	KY979852	KY979935
***N. ferruginea***	**G 00127285**	KY964330	KY964331	n/a	n/a
***N. garlbiwalawarda***	**BRIP 69595**	MN567686	n/a	MN604937	n/a
*N. garlbiwalawarda*	BRIP 69594	MN567685	n/a	MN604936	n/a
*N. garlbiwalawarda*	BRIP 69587	MN567684	n/a	MN604935	n/a
***N. ginkgonis***	**KUMCC 18-0139**	PZ575572	PZ575573	PZ583463	PZ583462
*N. gossypiicola*	CBS 377.67	GU237845	GU238079	KT389658	GU237611
*N. gossypiicola*	UTHSC DI16-294	LT592943	n/a	LT593082	LT593012
***N. infossa***	**CBS 123395**	MH863295	GU238089	KT389659	FJ427135
*N. infossa*	CBS 123394	FJ427024	GU238088	MN983642	FJ427134
***N. infuscata***	**CBS 121931**	MN973559	MN943766	MT018203	MT005662
*N. juglandis*	CGMCC 3.23563	MZ519403	n/a	MZ502664	MZ570583
*N. juglandis*	CGMCC 3.23564	MZ519404	n/a	MZ502665	MZ570584
***N. juglandis***	**CGMCC 3.23565**	MZ519405	n/a	MZ502666	MZ570585
***N. macrospora***	**CBS 140674**	LN880536	LN880537	LT593073	LN880539
***N. multilocularis***	**AUMC-H-0002.17**	n/a	KY996744	n/a	n/a
***N. naiawu***	**BRIP 69583**	MN567687	n/a	MN604938	n/a
***N. ngayawang***	**BRIP 69582**	MN567688	n/a	MN604939	n/a
***N. nullicana***	**CPC 32330**	MG386054	MG386107	MG386143	MG386165
***N. platycladi***	**CGMCC 3.27069**	PP600101	n/a	PP595977	PP595984
*N. platycladi*	CGMCC 3.27071	PP600103	n/a	PP595979	PP595986
*N. platycladi*	CGMCC 3.27070	PP600102	n/a	PP595978	PP595985
***N. populi***	**1_10**	PQ462358	n/a	PQ473512	PQ473515
***N. prosopidis***	**CBS 136415**	KF777149	KF777204	n/a	n/a
*N. prosopidis*	CPC 21701	KF777150	KF777205	n/a	n/a
*N. prosopidis*	CPC 21705	KF777152	KF777206	n/a	n/a
***N. pruni***	**MFLUCC 18-1601**	MH827005	MH827026	MH853662	MH853669
*N. quercina*	CBS 633.92	MH862380	EU754127	KT389657	GU237609
***N. raii***	**MCC 1082**	MF664467	n/a	n/a	MF664468
*N. raii*	A189	MF664467	MG590069	n/a	MF664468
***N. spiraeae***	**CFCC 53928**	MN737833	MN737828	MN879292	MN879295
***N. taboriae***	**BRIP 71522a**	OP599628	n/a	OP627086	n/a
***N. variabilis***	**UTHSC DI16-285**	LT592939	LN907428	LT593078	LT593008
*Phoma herbarum*	CBS 615.75	FJ427022	KF251715	KP330420	KF252703

Bayesian inference (BI) was analyzed by MrBayes on XSEDE v. 3.2.7a via the online web portal CIPRES Science Gateway v. 3.3, with two parallel runs. The best-fit model of nucleotide substitution was determined by MrModeltest v. 2.3 (Nylander 2004). Six simultaneous Markov chains were run for 2,000,000 generations but stopped automatically when the critical value for the topological convergence diagnostic reached below 0.01. Trees were sampled every 100^th^ generation. The initially sampling trees estimated by Tracer v. 1.7 (Rambaut et al. 2018) were treated as burn-in phase and discarded. The posterior probabilities in the majority rule consensus tree were then calculated by using the post burn-in phase of the remaining trees. ML analysis was implemented by the Randomized Axelerated Maximum Likelihood (RAxML) in RAxML-HPC v.8 on the XSEDE (8.2.12) tool via the online web portal CIPRES Science Gateway v. 3.3 (Miller et al. 2010) using default settings but adjusted with the GTR + GAMMAI model of nucleotide substitution and 1,000 rapid bootstrapping.

Phylograms were visualized by FigTree v1.4.0 program (Rambaut and Drummond 2012) and edited in Microsoft Office PowerPoint 2019 (Microsoft Inc., Redmond, WA, USA). Photographic trees and morphological photographic plates were edited by Adobe Photoshop CS6 Extended version 10.0 software (Adobe Systems Inc., San Jose, CA, USA). The nucleotide sequences of the new taxa were registered for the GenBank accession numbers.

## Results

### Phylogenetic analyses

In this study, two phylogenetic analyses were conducted to clarify the phylogenetic placements of three new species within the *Didymella* (Analysis 1), and *Nothophoma* (Analysis 2), as follows:

#### Analysis 1

**Figure 1.**
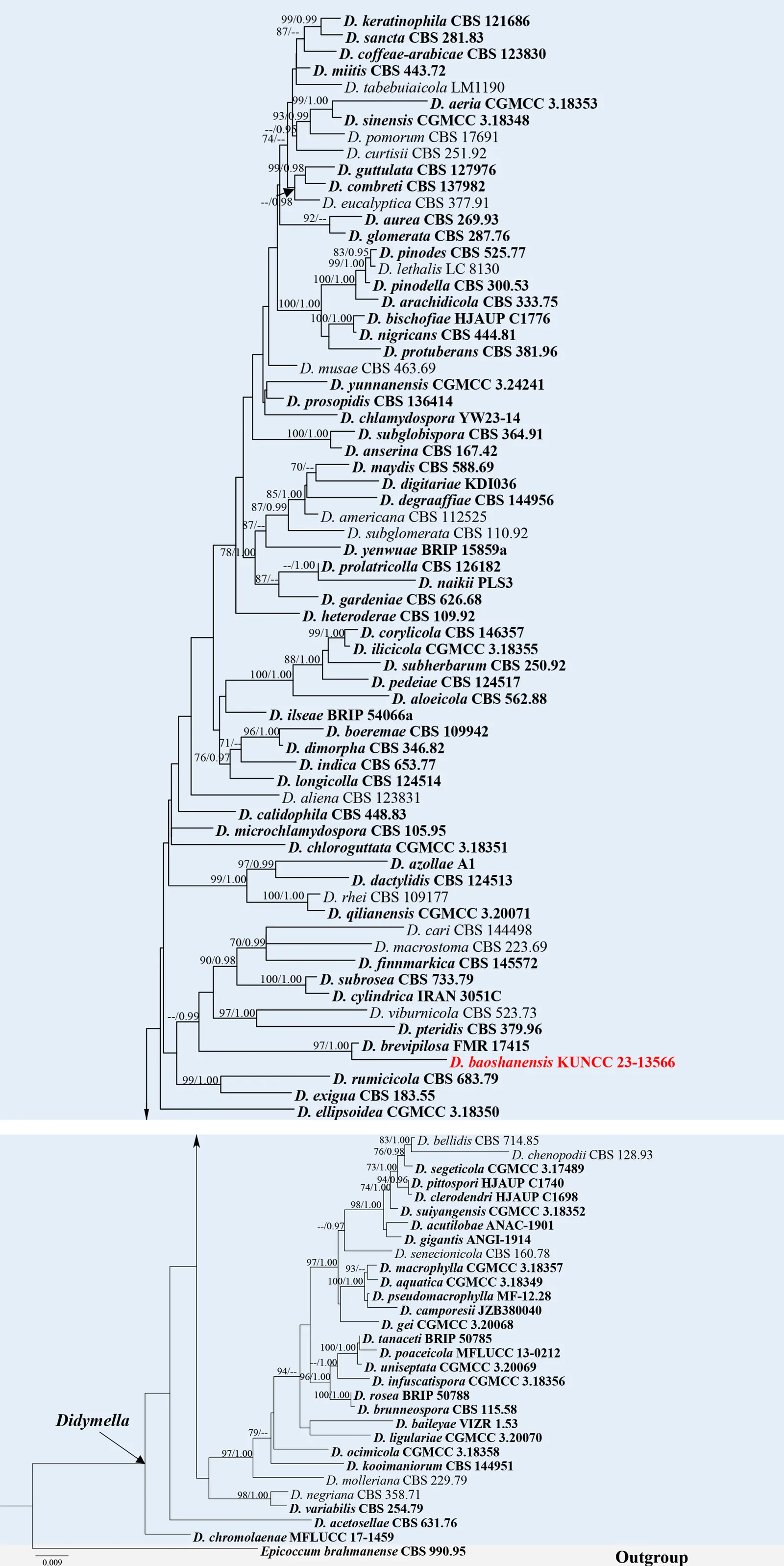
RAxML phylogram of the best-scoring ML consensus tree of *Didymella* species, based on the concatenated ITS, LSU, *rpb2*, and *tub2* sequence dataset. ML bootstrap support ≥ 70% (left) and Bayesian posterior probability values ≥ 0.95 (right) are given at the nodes (MLBS/BYPP). The tree is rooted to *Epicoccum brahmanense* (CBS 990.95). Ex-type strains are in bold, and the new species is indicated in red bold.

The phylogenetic tree of *Didymella* was constructed based on the concatenated ITS, LSU, *rpb2*, and *tub2* sequence dataset. A total of 97 strains of *Didymella* species (*Didymellaceae*) were included, with *Epicoccum brahmanense* (CBS 990.95) designated as the outgroup. The aligned dataset contained 2,412 characters (ITS: 1–504 bp, LSU: 505–1,466 bp, *rpb2*: 1,467–2,064 bp, and *tub2*: 2,065–2,412 bp), including gaps. The best-scoring RAxML tree was selected to represent the relationships among taxa, with a final likelihood value of -17721.763323 (Fig. 1). The matrix contained 703 distinct alignment patterns, with estimated base frequencies of A = 0.238184, C = 0.242209, G = 0.276073, T = 0.243534; substitution rates AC = 1.407157, AG = 6.806099, AT = 1.718222, CG = 0.856008, CT = 13.255759, GT = 1.000000; gamma distribution shape parameter α = 1.044481. For BI phylogenetic analysis, the optimal nucleotide substitution models were evaluated via the Akaike information criterion (AIC) implemented in MrModeltest: the SYM+I+G model was selected for the ITS locus, GTR+I+G for LSU, *rpb2*, and *tub2* loci. Six simultaneous Markov chains were run for 2,000,000 generations and trees were sampled every 100^th^ generation. The first 25% of trees were discarded as the burn-in phase of the analyses, and the remaining trees were used for calculating posterior probabilities in the majority rule consensus tree, of which the final average standard deviation of split frequencies at the end of total MCMC generations was 0.009871.

Phylograms inferred from ML and BI analyses show a similarity in tree topologies and are not significantly different. In the present study (Fig. 1), the phylogenetic affinities of many *Didymella* species are not well-resolved, including *D. acetosellae* (CBS 631.76), *D. acutilobae* (ANAC-1901), *D. aliena* (CBS 123831), *D. baileyae* (VIZR 1.53), *D. calidophila* (CBS 448.83), *D. camporesii* (JZB380040), *D. cari* (CBS 144498), *D. chlamydospora* (YW23-14), *D. chloroguttata* (CGMCC 3.18351), *D. chromolaenae* (MFLUCC 17-1459), *D. degraaffiae* (CBS 144956), *D. ellipsoidea* (CGMCC 3.18350), *D. gei* (CGMCC 3.20068), *D. gigantis* (ANGI-1914), *D. heteroderae* (CBS 109.92), *D. ilseae* (BRIP 54066a), *D. ligulariae* (CGMCC 3.20070), *D. macrostoma* (CBS 223.69), *D. microchlamydospora* (CBS 105.95), *D. miitis* (CBS 443.72), *D. musae* (CBS 463.69), *D. ocimicola* (CGMCC 3.18358), *D. poaceicola* (MFLUCC 13-0212), *D. prosopidis* (CBS 136414), *D. protuberans* (CBS 381.96), *D. pseudomacrophylla* (MF-12.28), *D. subherbarum* (CBS 250.92), *D. tabebuiaicola* (LM1190), *D. tanaceti* (BRIP 50785), and *D. yunnanensis* (CGMCC 3.24241). While phylogenetic placements of *D. aquatica* (CGMCC 3.18349), *D. aurea* (CBS 269.93), *D. coffeae-arabicae* (CBS 123830), *D. digitariae* (KDI036), *D. gardenia* (CBS 626.68), *D. glomerata* (CBS 287.76), *D. indica* (CBS 653.77), *D. kooimaniorum* (CBS 144951), *D. macrophylla* (CGMCC 3.18357), *D. maydis* (CBS 588.69), and *D. yenwuae* (BRIP 15859a) are well-resolved in ML analysis, with significant support value (up to 70% MLBS), but not support in BI analysis. In contrast, phylogenetic placements of *D. curtisii* (CBS 251.92), *D. eucalyptica* (CBS 377.91), *D. infuscatispora* (CGMCC 3.18356), *D. naikii* (PLS3), *D. prolatricolla* (CBS 126182), and *D. senecionicola* (CBS 160.78) are not well-resolved in ML analysis, but form well-resolved subclade with closely related taxa in BI analysis, with significant support value (up to 0.95 BYPP). The new species, *D. baoshanensis* (KUNCC 23-13566) forms a distinct sister lineage to *D. brevipilosa* (FMR 17415, ex-type) with high support value (97% MLBS and 1.00 BYPP; Fig. 1). These two species form a basal subclade, and cluster with *D. pteridis* (CBS 379.96), *D. viburnicola* (CBS 523.73), *D. cylindrica* (IRAN 3051C), *D. subrosea* (CBS 733.79), *D. finnmarkica* (CBS 145572), *D. macrostoma* (CBS 223.69) and *D. cari* (CBS 144498), respectively.

#### Analysis 2

**Figure 2.**
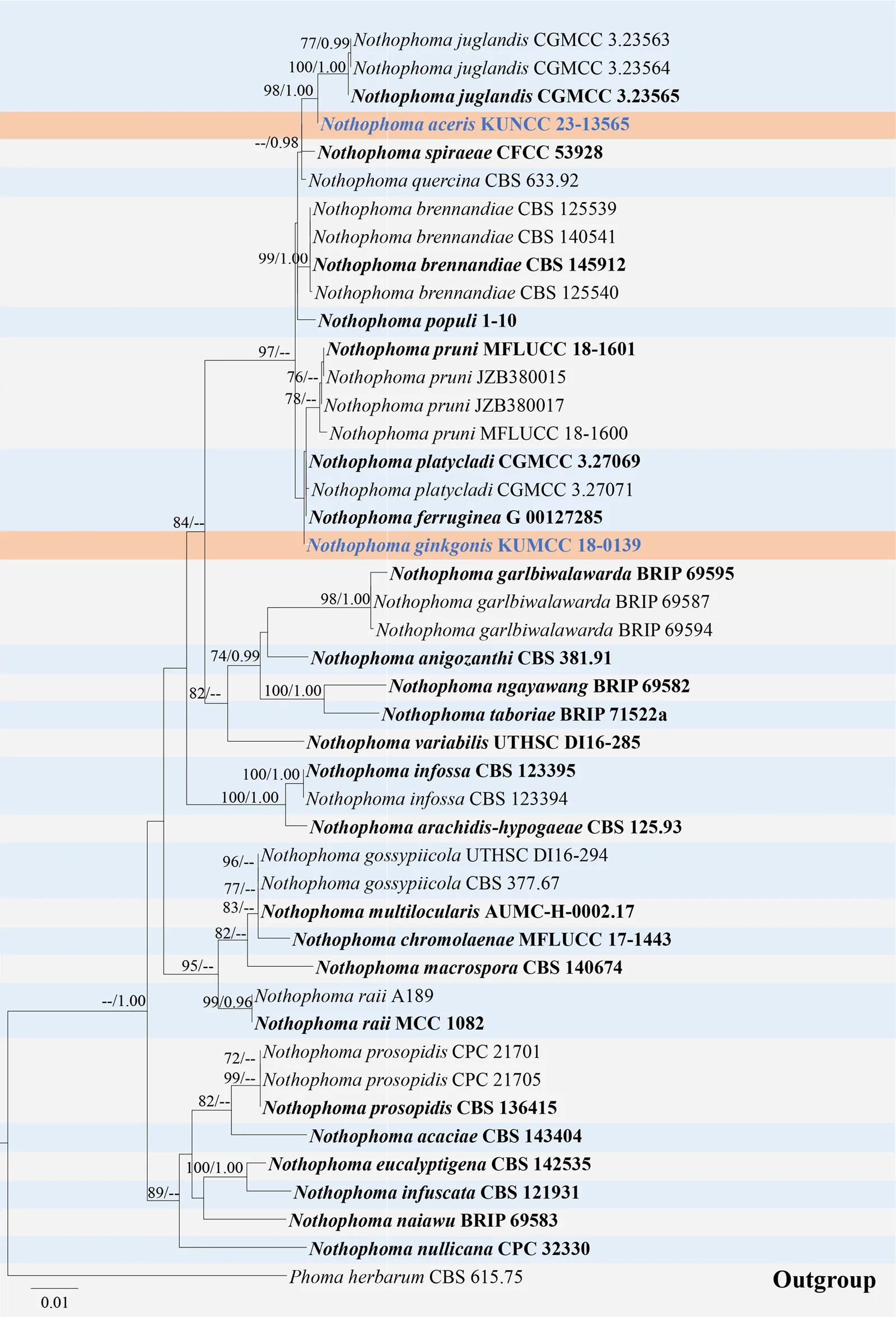
Phylogram of the best-scoring ML consensus tree of *Nothophoma* species based on a combined ITS, LSU, *rpb2*, and *tub2* sequence dataset. ML bootstrap values ≥ 70% (left) and Bayesian posterior probability ≥ 0.95 (right) are given at the nodes (MLBS/BYPP). The tree is rooted to *Phoma herbarum* (CBS 615.75). Ex-type strains are in bold, and new species are indicated in blue bold, highlighted by an orange box.

The phylogenetic tree of *Nothophoma* was constructed using sequence data from ITS, LSU, *rpb2*, and *tub2* of all *Nothophoma* species (*Didymellaceae*). A total of 45 strains were included, with one strain of *Phoma herbarum* (CBS 615.75) as the outgroup. The aligned dataset contained 2,723 characters (ITS: 1–547 bp, LSU: 548–1,510 bp, *rpb2*: 1,511–2,356 bp, and *tub2*: 2,357–2,723 bp), including gaps. The best-scoring RAxML tree was selected to represent the relationships among taxa, with a final likelihood value of -8027.380853 (Fig. 2). The matrix contained 605 distinct alignment patterns, with estimated base frequencies of A = 0.238713, C = 0.245622, G = 0.272314, T = 0.243351; substitution rates AC = 1.087703, AG = 4.396005, AT = 1.258730, CG = 0.529065, CT = 11.187075, GT = 1.000000; gamma distribution shape parameter α = 0.546894. For Bayesian inference phylogenetic analysis, the optimal nucleotide substitution models were determined via the AIC implemented in MrModeltest: the SYM+I+G model was selected for the ITS locus, GTR+I for LSU, GTR+I+G for *rpb2*, and HKY+I for *tub2*. Six simultaneous Markov chains were run for 2,000,000 generations and trees were sampled every 100^th^ generation. The first 25% of trees were discarded as the burn-in phase of the analyses, and the remaining trees were used for calculating posterior probabilities in the majority rule consensus tree, of which the final average standard deviation of split frequencies at the end of total MCMC generations was 0.008501.

Phylograms inferred from ML and BI analyses show a similarity in tree topologies and are not significantly different in the present study (Fig. 2). Multi-locus phylogenetic analyses demonstrated that the phylogenetic affinities of many *Nothophoma* species are not well-resolved, including *N. anigozanthi* (CBS 381.91), *N. ferruginea* (G 00127285), *N. ginkgonis* (KUMCC 18-0139), *N. naiawu* (BRIP 69583), *N. platycladi* (CGMCC 3.27069 and CGMCC 3.27071), *N. populi* (1-10), *N. quercina* (CBS 633.92), and *N. spiraeae* (CFCC 53928). Whereas the phylogenetic placements of *N. acaciae* (CBS 1434404), *N. chromolaenae* (MFLUCC 17-1443), *N. gossypiicola* (UTHSC DI 16-294 and CBS 377.67), *N. macrospora* (CBS 140674), *N. multilocularis* (AUMC-H-0002.17), *N. prosopidis* (CPC 21701, CPC 21705 and CBS 136415), *N. pruni* (MFLUCC 18-1601, JZB380015, JZB380017 and MFLUCC 18-1600), and *N. variabilis* (UTHSC DI 16-285) are well-resolved in ML analysis, with significant support value (up to 70% MLBS), but no support in BI analysis. The new species, *N. aceris* (KUNCC 23-13565), formed a distinct lineage sister to the taxon identified as *N. juglandis* (CGMCC 3.23563, CGMCC 3.23564, and CGMCC 3.23565) with high support values (98% MLBS and 1.00 BYPP). While *N. ginkgonis* (KUMCC 18-0139) form a distinct branch as basal of an unresolved subclade, comprising *N. ferruginea* (G 00127285), *N. platycladi* (CGMCC 3.27069 and CGMCC 3.27071), and *N. pruni* (MFLUCC 18-1601, JZB380015, JZB380017 and MFLUCC 18-1600).

### Taxonomy

#### Didymella baoshanensis

Taxon classificationFungiPleosporalesDidymellaceae

Phookamsak, H.B. Jiang, Bhat & Suwannar. sp. nov.

C00BDC69-D28D-5C8F-B18A-E7D32BA5208F

Index Fungorum: IF905778

Fig. 3

##### Etymology.

The specific epithet “*baoshanensis*” refers to the city, Baoshan City (Yunnan, China), where the holotype was collected.

##### Holotype.

KUN-HKAS 102221.

##### Description.

***Saprobic*** on dead rachis of an unidentified fern. ***Sexual morph*: *Ascostromata*** 250–950 µm long, bi- to multi-loculate (up to 5 locules), clustered in groups, semi-immersed to erumpent through host tissue, elongated, ellipsoidal to lenticularis, visible as black lines, dull, cracked on host surface, glabrous, each with an inconspicuous papilla. ***Ascomata*** 130–220 μm high (including ostiolar papilla), 120–210 µm diam., subglobose to ampulliform, sometimes irregular in shape, with individual, centrally located, narrowly rounded to truncate ostiole perched on a minute papillate canal. ***Peridium*** 6–35 μm wide, of equal thickness, composed of 3–6 layers of flattened, pseudoparenchymatous cells, with inner layers and layers in between the locules composed of hyaline, flattened cells, arranged in *textura angularis* to *textura prismatica*, with outer layers composed of brown to dark brown cells of *textura angularis*. ***Hamathecium*** composed of dense, 2.5–5.5 μm wide, septate, broadly cellular, filamentous pseudoparaphyses, constricted at septa, tapering towards apex, anastomosed above the asci, embedded in a hyaline gelatinous matrix. ***Asci*** (63.5–)75–90(–100) × (14–)16–19(–22) μm (*x̄* = 82 × 18 μm, *n* = 30; L/W = 3.9–5.6), 8-spored, bitunicate, fissitunicate, cylindrical to subcylindrical-clavate, subsessile to short pedicellate, apically rounded, with subconical ocular chamber clearly visible at immature state. ***Ascospores*** (14–)15–18(–21.5) × (6–)7–9(–10) μm (*x̄* = 17.4 × 8.1 μm, *n* = 50; L/W = 1.7–2.6), uni- to bi-seriately overlapping, fusiform, 1-septate, slightly constricted at septum, acute at upper end, rounded at lower end, hyaline, smooth-walled, with small guttules, sometimes slightly rough at surface, lacking a mucilaginous sheath. ***Asexual morph***: Undetermined.

**Figure 3.**
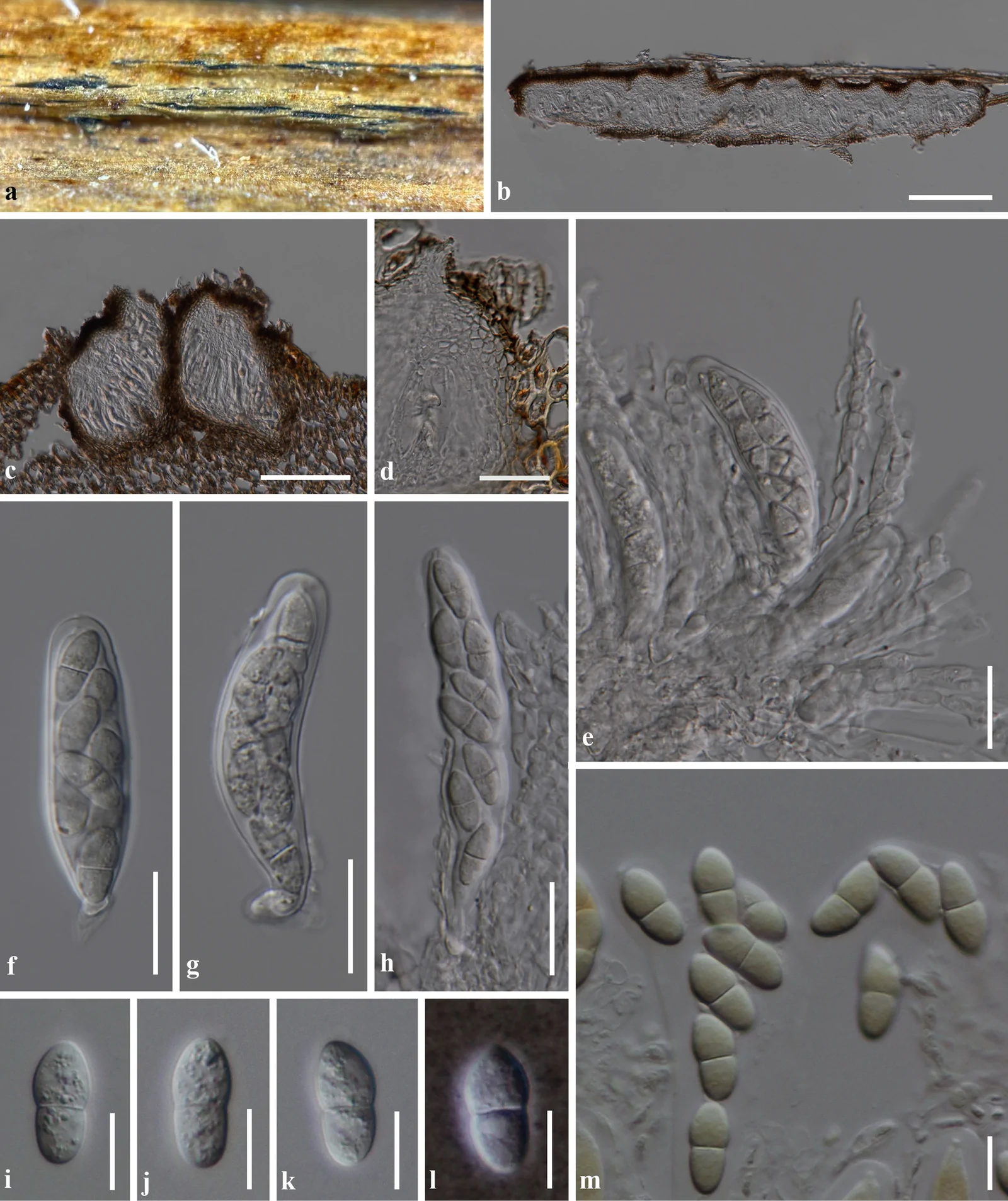
*Didymella baoshanensis* (KUN-HKAS 102221, holotype). **a**. The appearance of ascostromata on the host surface; **b, c**. Section of ascostoma; **d**. Section of peridium; **e**. Asci embedded in cellular pseudoparaphyses; **f–h**. Asci (notes: h = fissitunicate); **i–m**. Ascospores (notes: l = ascospore stained with Indian ink, m = ascospores stained with Melzer’s reagent). Scale bars: 100 µm (**b, c**); 50 µm (**d**); 20 µm (**e–h**); 10 µm (**i–m**).

##### Culture characteristics.

Colonies on PDA reaching 45–50 mm diam. after 2 weeks at room temperature (25–27 °C), dense, circular, flattened to slightly raised, fluffy to floccose, with a smooth surface and entire margin; surface initially white, becoming zonate, greenish olivaceous at margin, white to cream at center. Reverse white to cream at margin, dark brown to black in the middle, becoming pale reddish-brown towards the center, zonate. Pigment not produced on PDA. Sporulation not produced after 8 weeks.

##### Material examined.

China • Yunnan Province, Baoshan, Shuizai, Dawazi mountain, on dead hanging rachis of an unidentified fern, 22 October 2015, I.D. Goonasekara, BS009 (KUN-HKAS 102221, ***holotype***), ex-type living culture KUNCC 23-13566; *ibid*., KUMCC 18-0146.

##### Notes.

Based on NCBI nucleotide BLAST search of ITS region, the closest match of *Didymella baoshanensis* is *Didymella* sp. isolate 720-4 with 100% similarity (Identities = 479/479, with no gap). The species is also similar to *Didymella* sp. isolate D18L-19Pi with 99.79% similarity (Identities = 483/484, with no gap), *Epicoccum huancayense* (N 22) with 99.78% similarity (Identities = 463/464, with 1 gap), *Epicoccum* sp. isolate CB22-132 with 99.59% similarity (Identities = 482/484, with no gap), and *D. brevipilosa* (CBS 149049) with 98.97% similarity (Identities = 479/484, with no gap). The nucleotide BLAST search of *tub2* indicated that *D. baoshanensis* is similar to *Didymella* sp. (strains 720-4 and N84) with 99.70% and 99.64% similarities (Identities = 332/333 and 278/279, with no gap), respectively. Phylogenetically, *D. baoshanensis* formed a sister branch with the type strain of *D. brevipilosa* (FMR 17415) with high support values (97% MLBS and 1.00 BYPP; Fig. 1).

Morphologically, *Didymella baoshanensis* could not be compared with *D. brevipilosa* because they are represented by different morphs (Magaña-Dueñas et al. 2021; Crous et al. 2023). Besides, *D. brevipilosa* was isolated from plant debris submerged in freshwater in Spain and occurred on buds of *Abies balsamea* (*Pinaceae*) in Canada. Whereas *D. baoshanensis* was isolated from dead standing rachis of an unidentified fern in Yunnan (China). Notably, *Didymella* sp. strain 720-4 found as a pathogens casing leaf and stem blight symptom on bracken fern (*Pteridium aquilinum*) in South Korea (Lee et al. 2019). However, this isolate was not formally introduced and validated. Hence, the new species *D. baoshanensis* is introduced herein based on multi-locus phylogenetic evidence and the host association and habitats.

#### Nothophoma aceris

Taxon classificationFungiPleosporalesDidymellaceae

Phookamsak, Bhat, H.B. Jiang & Suwannar. sp. nov.

4555E41C-BB83-5603-B2DB-99DC794B9B70

Index Fungorum: IF905779

Fig. 4

##### Etymology.

The specific epithet “*aceris*” refers to the host, *Acer palmatum*, of which the holotype was collected.

##### Holotype.

KUN-HKAS 102216.

##### Description.

***Saprobic*** on dead aerial twigs of *Acer palmatum*, visible as pale to black knots or irregular cracked lines on host surface. ***Sexual morph***: Undetermined. ***Asexual morph*: *Conidiomata*** 80–115 µm high, 110–165 µm diam., pycnidial, immersed to erumpent, solitary, scattered to clustered, globose to subglobose, uni- to bi-loculate, becoming stromatic, brown to dark brown, glabrous, ostiolate, mostly apapillate, occasionally with a minute papilla. ***Pycnidial wall*** 8–25 µm wide, thin-walled of equal thickness, sometimes slightly thicker near apex, composed of 1–3(–6) layers of brown to dark brown pseudoparenchymatous cells, arranged in *textura angularis*. ***Conidiophores*** reduced to conidiogenous cells. ***Conidiogenous cells*** (3–)4–7 × 5–7 µm (*x̄* = 5 × 5.3 μm, *n* = 30; L/W = 0.57–1.40), holoblastic, phialidic, hyaline, ampulliform or somewhat isodiametric, unbranched, indeterminate, smooth-walled, sometimes percurrently proliferating at the same level 1–2 times. ***Conidia*** 5–7 × 4–5.5 µm (*x̄* = 6.1 × 4.6 μm, *n* = 50; L/W = 0.91–1.75), initially hyaline, later becoming brown to dark brown, subglobose or ovoid to obovoid, aseptate, thick- and smooth-walled, non-guttulate.

##### Culture characteristics.

Colonies on PDA reaching 40–42 mm diam. after 4 weeks at room temperature (20–27 °C), medium dense, circular, with a smooth surface and entire margin, flattened to slightly raised, fluffy to floccose; from above white to cream slightly radiating outwards colony with concentric rings; from below, white to cream. Pigment not produced on PDA. Sporulation not produced after 8 weeks.

##### Material examined.

China • Yunnan Province, Kunming, Panlong, Ciba, on dead aerial twigs of *Acer palmatum* (*Aceraceae*), 28 November 2015, R. Phookamsak, COE007, (KUN-HKAS 102216, ***holotype***), ex-type living culture KUNCC 23-13565; • *ibid*., KUMCC 18-0140.

##### Notes.

Based on NCBI nucleotide BLAST search of ITS sequence, the closest hits of *Nothophoma aceris* (KUNCC 23-13565) are *N. quercina* (isolate XHHB-2-1, KACC49903, DMLJXB-1-2-2, DWXB-1-1-1, ZJXB-2-3-2, BSXB-2-3-1, XJAU HC230-1-1, ZJXB-2-4, JZB380139, DWXB-1-1-2, WFH202105040, XHHB-1-1, DMLJDB-1-2-1, BSBM-1-1, XHHB-2, and DWDB-X-1-1), *Coniothyrium* sp. SS-2014 (isolate SB09), *Nothophoma* sp. (strain MEND-F-0791, isolate RTB-2021-036, and MEND-F-0792), *Epicoccum* sp. (isolate E575, and E387), *Didymellaceae* sp. (isolate 3.3T/1, FL-M-3, and DSM100405_DF10_RLCS12), *Nothophoma spiraeae* (strain CFCC 53930, and CFCC 53929), *Pleosporales* sp. (isolate UASWS2126), *Ascochyta medicaginicola* (isolate TA63-1), *Phoma* sp. (isolate BB17-10-1), *Microsphaeropsis olivacea* (isolate XJAU 3745-1), *Peyronellaea glomerata* (strain 10_4_M13F_C2J) and others with 99.80% similarity (Identities = 503/504 with 1 gap). In *rpb2* nucleotide BLAST search, the closest hits of *N. aceris* (KUNCC 23-13565) are *Nothophoma* sp. (strain GBB18) with 100% similarity (Identities = 1071/1071 with no gap), *N. quercina* (isolate ZQ202004002), and *N. quercina* (isolate PDB-1) with 100% similarity (Identities = 884/884 with no gap). *Nothophoma aceris* (KUNCC 23-13565) is also similar to *N. quercina* (GenBank no: MN900955) with 99.90% similarity (Identities = 1033/1034 with 1 gap), *N. quercina* (isolate E96) with 99.90% similarity (Identities = 993/994 with no gap), and *N. quercina* (MFLUCC 18-1550) with 99.89% similarities (Identities = 947/948 with 1 gap). In *tub2* nucleotide BLAST search, the closest hits of *N. aceris* (KUNCC 23-13565) are *N. pruni* (isolates JZB380092, and JZB380089) with 100% similarity (Identities = 328/328 with no gap), *N. pruni* (isolates JZB380091, JZB380090, and JZB380088) with 100% similarity (Identities = 326/326 with no gap), and is similar to *N. quercina* (strains JZB380136, JZB380138, JZB380140, JZB380141, JZB380142, and JZB380144) with 99.70% similarity (Identities = 327/328 with no gap). Phylogenetic analyses based on a concatenated ITS, LSU, *rpb2*, and *tub2* sequence dataset demonstrated that *N. aceris* (KUNCC 23-13565) is closely related to the invalidly published name *N. juglandis* (CGMCC 3.23563, CGMCC 3.23564, and CGMCC 3.23565). *Nothophoma aceris* also clustered with *N. spiraeae* (strain CFCC 53928; ex-type) and *N. quercina* (strain CBS 633.92) with significant support in BI analysis (0.98 BYPP; Fig. 2), but low support in ML analysis.

Species of *Nothophoma* are difficult to distinguish based on morphological characteristics alone. *Nothophoma aceris* is morphologically similar to the invalidly published name *N. juglandis* in having pycnidial, dark brown, globose conidiomata, phialidic, hyaline, smooth, ampulliform conidiogenous cells, and ovoid, aseptate conidia (Zhao et al. 2024). However, *N. aceris* can be distinguished from *N. juglandis* in having smaller conidiomata (80–115 × 110–165 µm, L/W = 0.48–1.05 vs. 285–600 × 240–480 µm, L/W = 0.59–2.50), which are globose to subglobose, uni- to bi-loculate, ostiolate, apapillate, whereas those of *N. juglandis* are solitary or confluent, globose to irregular, with papillate or elongated neck. Conidiogenous cells of *N. aceris* are slightly smaller than those reported for *N. juglandis* [(3–)4–7 × 5–7 µm, LW = 0.57–1.40 vs. 7–10.5 × 6–9 μm, L/W = 0.78–1.75]. In contrast, the conidial dimensions overlap between *N. aceris* and *N. juglandis* (5–7 × 4–5.5 µm, L/W = 0.9–1.8 vs. 5–8 × 4–6 µm, L/W = 0.83–2.00). Nevertheless, the conidia differ in morphology: *N. aceris* produces hyaline to brown or dark brown, subglobose or ovoid to obovoid, aseptate, thick- and smooth-walled, non-guttulate conidia, whereas *N. juglandis* produces hyaline to olivaceous buff, ovoid, oblong to ellipsoidal, smooth and thin-walled conidia, sometimes containing small guttules (Zhao et al. 2024). Furthermore, the two taxa also differ in host association and ecology. *Nothophoma aceris* was isolated from dead aerial twigs of *Acer palmatum*, whereas the fungus identified as *N. juglandis* was reported as a pathogen causing heart rot disease of walnut in northwest Beijing, China (Zhao et al. 2024).

**Figure 4.**
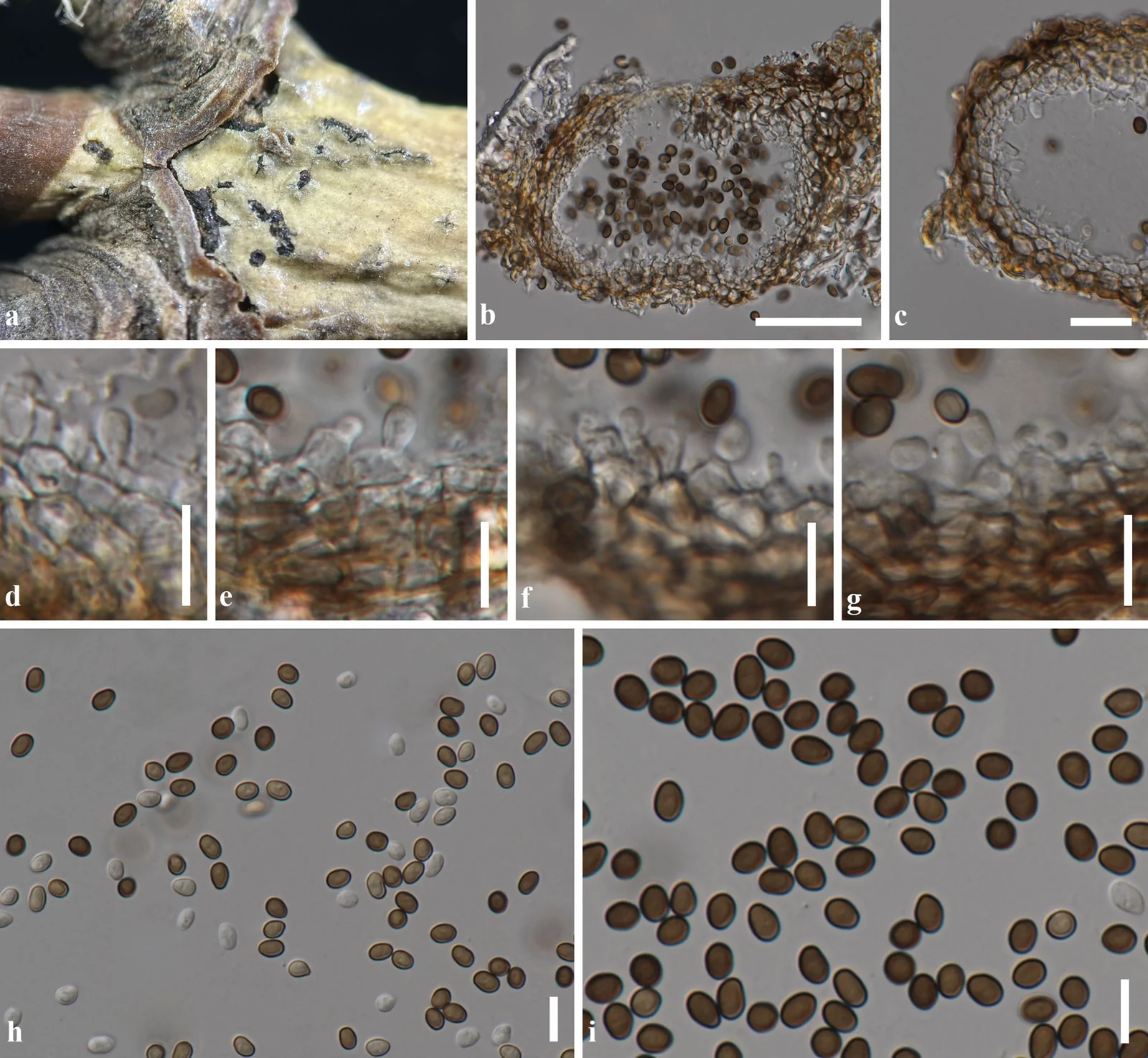
*Nothophoma aceris* (KUN-HKAS 102216, holotype). **a**. The appearance of conidiomata on the host surface; **b**. Section of pycnidial conidioma; **c**. Section of pycnidial wall; **d–g**. Conidiogenous cells bearing conidia; **h, i**. Conidia. Scale bars: 50 µm (**b**); 20 µm (**c**); 10 µm (**d–i**).

*Nothophoma aceris* resembles *N. quercina* (strains MFLUCC 18-1550, ZQ202004002, and XJAKS05) in sharing the size range and characters of conidiomata, conidiogenous cells, and conidia. However, *N. aceris* can be distinguished from *N. quercina* (strain MFLUCC 18-1550) in having slightly larger conidia (5–7 × 4–5.5 µm, L/W = 0.91–1.75 vs. 5.1–6.5 × 3.3–4.5, L/W = 1.13–1.97 μm; Jayasiri et al. 2019). While *N. quercina* (strain ZQ202004002) has larger conidiomata (284–427 × 326–554 µm, L/W = 0.51–1.31 vs. 80–115 × 110–165 µm, L/W = 0.48–1.05) and conidia (5–8 × 4–6 µm, L/W = 0.83–2.00 vs. 5–7 × 4–5.5 µm, L/W = 0.91–1.75) (Zeng et al. 2022). Also, *N. quercina* (strain XJAKS05) has slightly smaller conidiomata (65–135 µm in diam.) and conidia (4.3–8.5 × 3–4.8 µm, L/W = 0.90–2.83) (Bai et al. 2016). Furthermore, *N. quercina* (strain MFLUCC 18-1550) was isolated from a decaying cone of *Keteleeria fortunei* (*Pinaceae*) while *N. quercina* (strain ZQ202004002) was isolated from leaf blight symptoms of *Magnolia coco* (Zeng et al. 2022) and *N. quercina* (strain XJAKS05) was isolated from brown spots of *Ziziphus jujuba* in China (Bai et al. 2016). *Nothophoma aceris* can be distinguished from *N. spiraeae* (strains CFCC 53928, and CFCC 53929) in having smaller conidiomata (80–115 × 110–165 µm, L/W = 0.48–1.05 vs. (145–)155–280(–300) × (120–)140–230(–250) μm, L/W = 0.67–2.00), larger conidia (5–7 × 4–5.5 µm, L/W = 0.91–1.75 vs. 5–6.5(–7) × (3–)3.5–4 μm, L/W = 1.25–2.33) and lacking papilla necks. While *N. spiraeae* has a distinctly elongated neck (Zhang et al. 2020). Therefore, *N. aceris* is introduced herein as a new species.

#### Nothophoma ginkgonis

Taxon classificationFungiPleosporalesDidymellaceae

Phookamsak, H.B. Jiang, Bhat & Suwannar. sp. nov.

E492644C-3093-5E2B-AD45-46419529A761

Index Fungorum: IF905780

Fig. 5

##### Etymology.

The specific epithet “*ginkgonis*” refers to the host, *Ginkgo biloba*, of which the holotype was collected.

##### Holotype.

KUN-HKAS 102215.

##### Description.

***Saprobic*** on dead aerial twigs of *Ginkgo biloba*, visible as small, black knots on the host surface. ***Sexual morph***: Undetermined. ***Asexual morph*: *Conidiomata*** 90–110 µm high, 120–170 µm diam., pycnidial, immersed to semi-immersed, slightly raised, becoming erumpent through host epidermis by protruding ostiole, solitary, scattered, subglobose to hemispherical or ellipsoidal, uni-loculate, brown, slightly dark brown to black at apex, glabrous, ostiolate, apapillate. ***Pycnidial wall*** (6–)10–20(–23.5) µm wide, with thin-walled cells of equal thickness, occasionally thicker at apex, composed of 2–3(–6) layers, of light brown to brown pseudoparenchymatous cells, arranged in a *textura angularis*, with outer layers composed of dark brown to black cells, with *textura globulosa* to *textura epidermoidea* cells at apex. ***Conidiophores*** reduced to conidiogenous cells. ***Conidiogenous cells*** (2–)4–5(–7) × (2.5–)3–5(–7) µm (*x̄* = 4.8 × 4.7 μm, *n* = 40; L/W = 0.4–2.8), holoblastic, phialidic, hyaline, ampulliform or somewhat isodiametric, unbranched, determinate, smooth-walled. ***Conidia*** 6–8 × (3.5–)4–5.5 µm (*x̄* = 6.9 × 4.8 μm, *n* = 50; L/W = 1.1–2.3), initially hyaline, becoming light brown to yellowish brown, subglobose to ellipsoidal, or obovoid, aseptate, smooth-walled, with small guttules inside.

**Figure 5.**
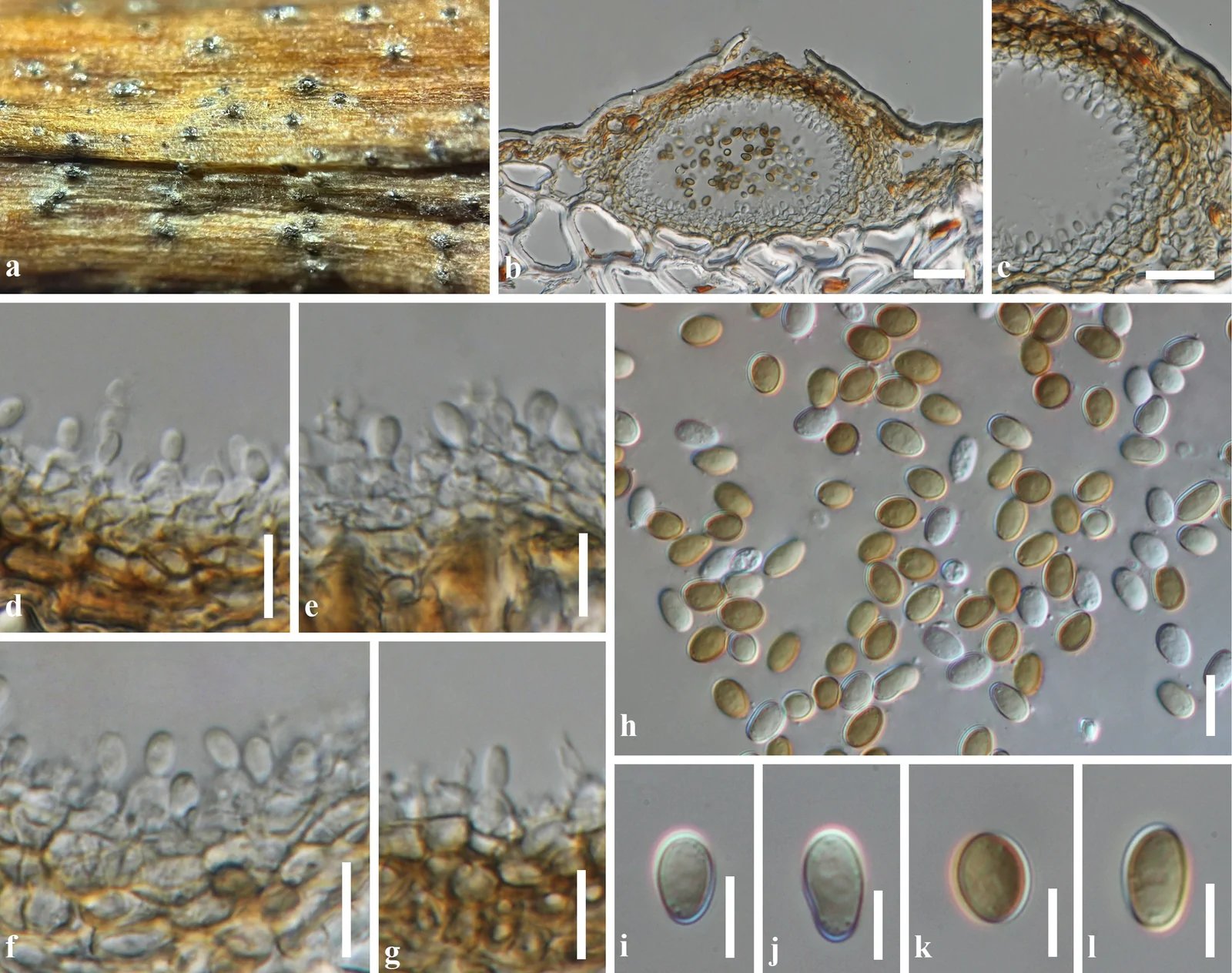
*Nothophoma ginkgonis* (KUN-HKAS 102215, holotype). **a**. The appearance of conidiomata on the host surface; **b**. Section of pycnidial conidioma; **c**. Section of pycnidial wall; **d–g**. Conidiogenous cells bearing conidia; **h–l**. Conidia. Scale bars: 50 µm (**b**); 20 µm (**c**); 10 µm (**d–h**); 5 µm (**i–l**).

##### Culture characteristics.

Colonies on PDA reaching 45–48 mm diam. after two weeks at room temperature (20–27 °C), dense, circular, flattened to slightly raised, fluffy to floccose, with a smooth surface and entire margin; from above initially white to cream, becoming dirty grey with age; from below white to cream at the margin, becoming dark brown to black towards the center. Pigment not produced on PDA. Sporulation not produced after 8 weeks.

##### Material examined.

China • Yunnan Province, Kunming, Panlong, Ciba, on dead aerial twigs of *Ginkgo biloba* (*Ginkgoaceae*), 29 November 2015, R. Phookamsak, COE004, (KUN-HKAS 102215, ***holotype***), ex-type living culture KUMCC 18-0139.

##### Notes.

Based on NCBI nucleotide BLAST search of ITS sequence, the closest hits of *Nothophoma ginkgonis* (KUMCC 18-0139) are *Coniothyrium pyrinum* (isolate Z196), *Didymellaceae* sp. (isolate XY11-12-2-3 and KQ5-21), *Paraconiothyrium fuckelii* (isolate 0312MAR24X2 and 0312MAR24X2) with 100% similarity (Identities = 510/510 with no gap). In *rpb2* nucleotide BLAST search, the closest hits of *N. ginkgonis* (KUMCC 18-0139) are *N. quercina* (GenBank no. PP228377, and isolate S11) with 99.72% similarity (Identities = 1064/1067 bp with 3 gaps, and 1060/1063 bp with 3 gaps, respectively). *Nothophoma ginkgonis* (KUMCC 18-0139) is also similar to *N. quercina* (isolate QJ-M-6) with 99.62% similarity (Identities = 1044/1048 with 1 gap) and *N. spiraeae* (culture VKM:F-5161) with 98.41% similarity (Identities = 1051/1068 with 4 gap). In *tub2* nucleotide BLAST search, the closest hits of *N. ginkgonis* (KUMCC 18-0139) are *N. pruni* (isolates JZB380088, JZB380090, and JZB380091) with 100% similarity (Identities = 328/328 with no gap), *N. pruni* (isolates JZB380089, and JZB380092) with 100% similarity (Identities = 326/326 with no gap), and is similar to *N. spiraeae* (culture VKM:F-5161) with 99.70% similarity (Identities = 332/333 with no gap), and *N. pruni* (isolate JZB380100) with 99.70% similarity (Identities = 328/329 with no gap). Phylogenetic analyses based on a concatenated ITS, LSU, *rpb2*, and *tub2* sequence dataset demonstrated that *N. ginkgonis* (KUMCC 18-0139) is closely related to *N. ferruginea* (G 00127285), *N. platycladi* (CGMCC 3.27069 and CGMCC 3.27071) and *N. pruni* (MFLUCC 18-1601, JZB380015, JZB380017 and MFLUCC 18-1600) (Fig. 2), although this relationship receives weak support in both the ML and BI analyses (36% MLBS and 0.54 BYPP; tree not shown).

*Nothophoma ginkgonis* (KUN-HKAS 102215) is morphologically similar to *N. platycladi* in having dark brown to black, pycnidial, semi-immersed, subglobose to hemispherical, glabrous conidiomata, phialidic, hyaline, ampulliform, smooth-walled conidiogenous cells, and hyaline, ellipsoidal, smooth-walled, aseptate conidia (Jiao et al. 2024). However, *N. ginkgonis* can be distinguished from *N. platycladi* in having smaller (90–110 × 120–170 µm, L/W = 0.53–0.92 vs. 301–545 × 236–456 μm, L/W = 0.66–2.31), apapillate conidiomata, slightly larger conidiogenous cells [(2–)4–5(–7) × (2.5–)3–5(–7) µm, L/W = 0.29–2.80 vs. 4.0–6.5 × 2.5–4.5 μm, L/W = 0.89–2.60], and larger (6–8 × (3.5–)4–5.5 µm, L/W = 1.09–2.29 vs. 4.7–6.4 × 2.8–4.3 μm, L/W = 1.09–2.29), light brown to yellowish brown, subglobose to ellipsoidal, or obovoid conidia. Whereas *N. platycladi* has semi-immersed or superficial, spherical to irregular shaped, mostly aggregated conidiomata, with papillate or elongated neck and hyaline but incidentally olivaceous buff conidia (Jiao et al. 2024). *Nothophoma ginkgonis* (KUN-HKAS 102215) resembles *N. pruni* (MFLU 18–2668, holotype) in having solitary, scattered, globose to irregularly shaped, black, ostiolate conidiomata, phialidic, hyaline, ampulliform conidiogenous cells, and hyaline, obovoid, aseptate, smooth-walled conidia. However, the conidia of *N. ginkgonis* are slightly shorter and broader (6–8 × (3.5–)4–5.5 µm, L/W = 1.09–2.29 vs. 4.8–8.5 × 2.7–3.9 µm, L/W = 1.23–3.15), and become light brown to yellowish-brown, subglobose to ellipsoidal, or obovoid at age whereas the conidia of *N. pruni* are hyaline, cylindrical to obovoid or oblong, with hyaline to buff conidial exudates (Chethana et al. 2019). Furthermore, *N. pruni* was found on diseased leaves of *Prunus avium* (*Rosaceae*) (Chethana et al. 2019) while *N. ginkgonis* was isolated from dead aerial twigs of *Ginkgo biloba* (*Ginkgoaceae*). Unfortunately, the morphological characteristics of *N. ginkgonis* (KUN-HKAS 102215) could not be compared with *N. ferruginea* (G 00127285) due to these two species represented in different morph (Zhang et al. 2021). Based on morphological comparison, multi-locus phylogenetic evidence and host associations, *N. ginkgonis* is introduced herein as a new species.

## Discussion

*Didymella* is one of the important genera in *Pleosporales* and contains several phytopathogenic species occurring on broad range of host plants (Chen et al. 2015, 2017, 2022; Keirnan et al. 2021; Luo et al. 2024; Wang et al. 2024; Liu et al. 2025). The majority of described *Didymella* species have been isolated as plant pathogens, endophytes, or saprobes spanning more than 40 plant families (Chen et al. 2017, 2022; Luo et al. 2024; Wang et al. 2024). However, our understanding of their diversity and distribution across other substrates remains highly constrained (Hou et al. 2020b). Recent investigations have recovered *Didymella* species from diverse environmental niches, including air, lake water, limestone in caves, inorganic materials (e.g., asbestos, cement and paint), saline-alkali soils, and oligotrophic environments as well as clinical specimens such as human cutaneous lesions, skin, toenails, and sputum (Aveskamp et al. 2010; Chen et al. 2015, 2017, 2022; Zhang et al. 2017; Valenzuela-Lopez et al. 2018; Hou et al. 2020a, 2020b; Luo et al. 2024). These findings strongly indicate that a vast number of novel *Didymella* taxa remain undocumented, particularly within non-vegetative ecological niches.

In the present study, the new species *Didymella baoshanensis* was isolated from dead hanging rachis of an unidentified fern. To date, there are only a few *Didymella* species reported from fern hosts, including *D. azollae*, *D. cylindrica*, *D. hyphenis*, *D. lophospora*, *D. prominula*, *D. pteridis*, and *D. sinensis* (Farr et al. 2026). Among these taxa, the phylogenetic affinities within *Didymella* have been resolved only for *D. azollae*, *D. cylindrica*, *D. pteridis*, and *D. sinensis* (Chen et al. 2015, 2017; Valenzuela-Lopez et al. 2018; Hou et al. 2020a; Boonmee et al. 2021; Ahmadpour et al. 2022; Farr et al. 2026; Li et al. 2026). In contrast, *D. hyphenis*, *D. lophospora*, and *D. prominula* lack the molecular data required to clarify their precise phylogenetic placement. Accordingly, *D. baoshanensis* formed a well-resolved subclade with *D. brevipilosa*, remaining phylogenetically distinct from all other known fern-associated *Didymella* species.

Species within the genus *Didymella* exhibit high morphological plasticity, rendering species delineation based solely on morphological traits highly challenging. Consequently, modern taxonomic frameworks rely heavily on molecular data to achieve accurate identification. Recent comprehensive taxonomic revisions have demonstrated that a multi-locus approach utilizing four molecular markers, viz. ITS, LSU (for generic level), *rpb2*, and *tub2* (for species-level), is generally effective for resolving phylogenetic relationships within *Didymella*. Among these, *rpb2* has emerged as the most robust single marker for both generic and species-level delineation (Chen et al. 2015, 2017, 2022; Valenzuela-Lopez et al. 2018; Hou et al. 2020a, 2020b; Luo et al. 2024). Nevertheless, the phylogenetic affinities of several *Didymella* species remain unresolved within the current multilocus phylogenetic analyses. To achieve better phylogenetic resolution and more definitive species boundaries, future investigations should incorporate additional protein-coding genes such as actin (*act*), calmodulin (*cal*), and translation elongation factor 1-α (*tef1-α*) alongside comparative whole-genome analyses.

*Nothophoma* is a well-established genus in *Didymellaceae* and is known for both sexual morph (e.g., *N. anigozanthi*, *N. chromolaenae*, and *N. ferruginea*) and coelomycetous asexual morph (Chen et al. 2015; Mapook et al. 2020; Zhang et al. 2021). Chen et al. (2015) introduced *Nothophoma* alongside eight other novel genera within the family *Didymellaceae*, including *Allophoma*, *Calophoma*, *Heterophoma*, *Neoascochyta*, *Neodidymelliopsis*, *Paraboeremia*, *Phomatodes* and *Xenodidymella* based on a polyphasic approach. While certain species have been reported to exhibit narrow host-specificity, such as *N. infossa* and *N. gossypiicola* (Aveskamp et al. 2010; Chen et al. 2015), other congeneric species were reported as endophytes, pathogens, or saprobes across diverse host plants and environmental niches, including garden soil (Chen et al. 2015; Liu et al. 2018; Hou et al. 2020a, 2020b; Nekrasov et al. 2022; Wang et al. 2023; Martino et al. 2025). In the present study, we introduced two new species, *N. aceris* and *N. ginkgonis*, isolated from dead aerial twigs of *Acer palmatum* and *Ginkgo biloba* in Yunnan, China. These discoveries expand both the documented host ranges and geographical distribution of *Nothophoma* in this region. Notably, several *Nothophoma* species have been reported as phytopathogens (Chethana et al. 2019; Wang et al. 2023; Martino et al. 2025); the potential pathogenicity of *N. aceris* and *N. ginkgonis* warrants further investigation.

In accordance with Nekrasov et al. (2022), *Nothophoma quercina* constitutes a species complex that encompasses *N. brennandiae*, *N. pruni*, *N. quercina*, and *N. spiraeae*. Subsequent phylogenetic analyses indicated that *N. ferruginea*, the invalidly published name *N. juglandis*, *N. platycladi*, and *N. populi* also cluster within this species-complex subclade (Jiao et al. 2024; Zhao et al. 2024; Guo et al. 2025). However, because molecular data are unavailable for the type specimen of *N. quercina*, the precise phylogenetic placement of this species remains unresolved. To address this taxonomic ambiguity, we propose our two novel species, namely *N. aceris* and *N. ginkgonis*, based on an integrative morpho-molecular approach in the present study. These two new species clustered within the *N. quercina* species-complex subclade. *Nothophoma aceris* formed a well-resolved sister lineage to the taxon identified as *N. juglandis*. However, *N. juglandis* was invalidly published by Zhao et al. (2024) (nom. inval., Arts 40.1, 40.8; Shenzhen Code) because its protologue did not satisfy the requirements for valid publication. Under Art. 40.1, the type of a new taxon at the rank of genus or below must be indicated, while Art. 40.8 requires that, for the name of a new species or infraspecific taxon published on or after 1 January 2019 of which the type is a culture, the protologue must include a statement that the culture is preserved in a metabolically inactive state (Turland et al. 2018). Therefore, the name *N. juglandis* is not validly published and requires formal validation before it can be treated as a validly published species name.

*Nothophoma ginkgonis* formed a distinct basal lineage relative to *N. ferruginea*, *N. platycladi* and *N. pruni*. The phylogenetic relationships among these taxa remain poorly resolved, consistent with previous studies (Jiao et al. 2024; Zhao et al. 2024; Guo et al. 2025). Notably, the sequence alignments of some *Nothophoma* species in this subclade contain only short overlapping regions because different primer sets were used for amplification of the *tub*2 and *rpb*2 loci and, in some cases, the quality of the assembled sequences. Consequently, nucleotide base comparison between the two new species and their closely related taxa could not be reliably performed in the present study. To improve phylogenetic resolution and facilitate robust species delimitation within *Nothophoma*, future investigations should incorporate *tub2*, *rpb2*, and additional protein-coding loci, such as actin (*act*), calmodulin (*cal*), and translation elongation factor 1-α (*tef1*-*α*), generated using standardized primer sets across taxa.

Yunnan Province has repeatedly been recognized as an important hotspot for fungal diversity in China, particularly for microfungi associated with dead plant materials and other underexplored substrates (Wanasinghe et al. 2024, 2025). Recent taxonomic studies of ascomycetes from Yunnan have continued to reveal novel species, new records, host associations, and poorly known fungal lineages from bamboo, woody litter, herbaceous plants, grassland ecosystems, and other plant-associated substrates (Dissanayake et al. 2024; Gao et al. 2024; Li et al. 2024; Luo et al. 2024; Han et al. 2025; Wanasinghe et al. 2025; Liu et al. 2026; Xu et al. 2026). These studies demonstrate that many fungal groups remain insufficiently sampled, especially on dead or senescent plant materials, which often serve as important reservoirs of hidden fungal diversity. In particular, studies on saprobic and plant-associated ascomycetes have shown that dead twigs, rachides, culms and other decomposing plant tissues are valuable substrates for discovering novel taxa (Dissanayake et al. 2024; Li et al. 2024; Wanasinghe et al. 2025). Therefore, the discovery of *Didymella baoshanensis*, *Nothophoma aceris* and *N. ginkgonis* from dead plant substrates in Baoshan and Kunming further supports the view that continued field surveys, combined with detailed morphological observations and multilocus phylogenetic analyses, are essential for documenting the hidden diversity of *Didymellaceae* and other ascomycetous fungi in Yunnan and beyond.

## Supplementary Material

XML Treatment for Didymella baoshanensis

XML Treatment for Nothophoma aceris

XML Treatment for Nothophoma ginkgonis

## Additional information

### Artificial Intelligence (AI) use

The authors accept full responsibility for the content of the manuscript, including the disclosure of any use of AI.

No AI tools were used in the preparation of this manuscript.

### Author ORCIDs

R. Phookamsak https://orcid.org/0000-0002-6321-8416

H. Jiang https://orcid.org/0000-0001-9655-4662

J. Li https://orcid.org/0009-0008-6088-2072

P. Chomnunti https://orcid.org/0000-0003-2989-1735

H. Qi https://orcid.org/0009-0002-9337-4206

D.J. Bhat https://orcid.org/0000-0002-3800-5910

J. Kumla https://orcid.org/0000-0002-3673-6541

J. Xu https://orcid.org/0000-0002-2485-2254

L.K.T. Al-Ani https://orcid.org/0000-0001-5138-0224

S. Lumyong https://orcid.org/0000-0002-6485-414X

N. Suwannarach https://orcid.org/0000-0002-2653-1913

### Data availability

All of the data that support the findings of this study are available in the main text.
